# Supplementary material for: Differences in Aroma Metabolite Profile, Microstructure, and Rheological Properties of Fermented Milk Using Different Cultures
Source: Foods. 2023 May 2;12(9):1875. doi: 10.3390/foods12091875 (PMC10178633; doi:10.3390/foods12091875)
Supplement: Supplementary file 1 [file foods-12-01875-s001.zip › foods-2335057-supplementary.pdf]

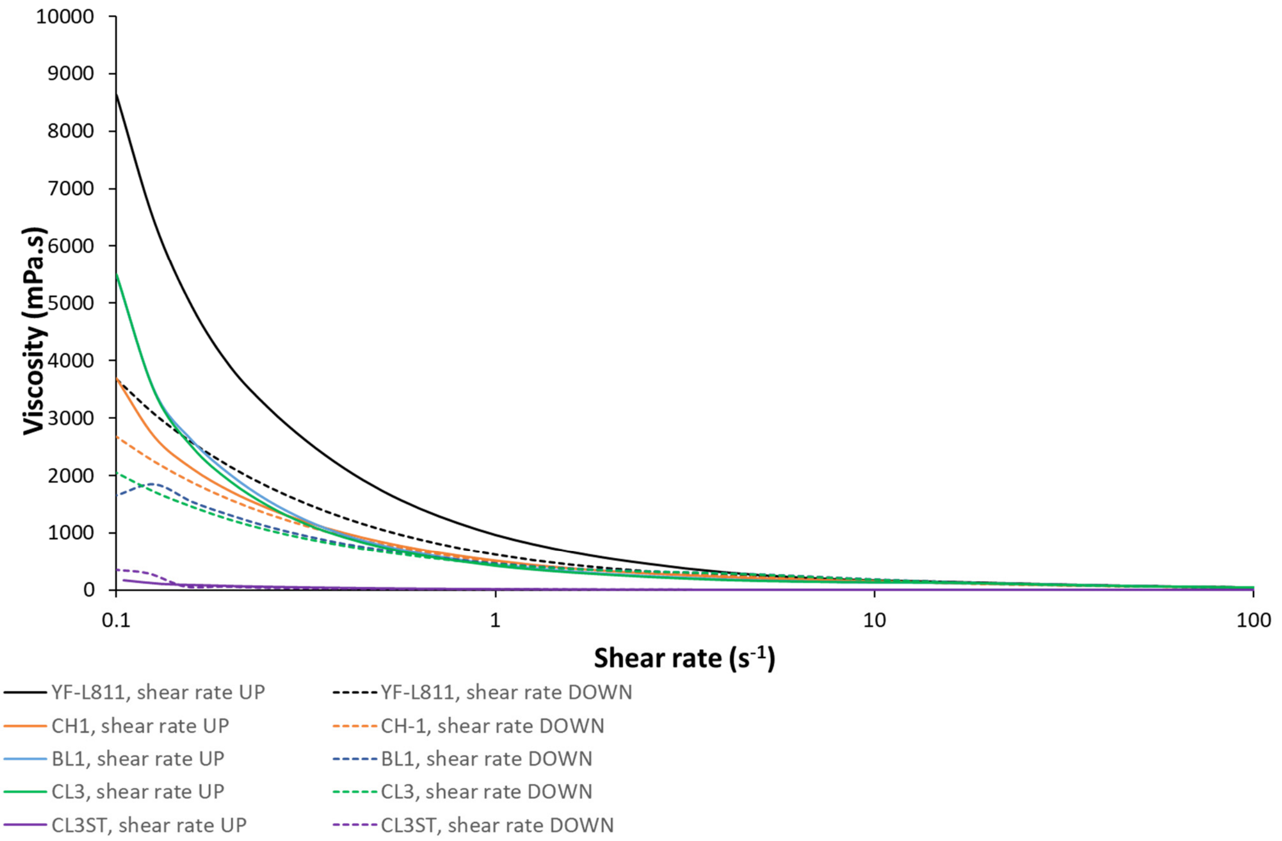

Figure S1. Viscosity as a function of shear rate of fermented milk samples. The continuous curves represent the increasing shear rate (upward curves) and discontinuous curves represent the decreasing shear rate (downward curves). Each data point is the average of six measurements (n=6).

Supplementary Table S1. Abundance of aroma metabolites in fermented milk samples. (Mean abundance ± standard deviation).

Compound	RIa	Ionsb	Fermented milk samples						
			BL1	CH-1	CL3	CL3ST	LH	ST	YF-L811
1,7_octadien-3-ol	1562	81	11278 ± 1355 c	31031 ± 2286 b	12670 ± 2508 c	12936 ± 1440 c	38858 ± 3256 a	29654 ± 1037 b	33977 ± 391 ab
1-butanol	1165	56	295789 ± 87041 a	34787 ± 29508 bc	15849 ± 8540 bc	221293 ± 115294 ab	175562 ± 167263 abc	13538 ± 7335 bc	12663 ± 6544 c
1-hydroxy propanone	1280	74	2931 ± 292 bc	1013 ± 507 d	2044 ± 231 c	2659 ± 266 bc	8718 ± 417 a	3685 ± 706 b	2448 ± 267 c
1-Octen-3-ol	1453	57	21010 ± 12505 c	47902 ± 28722 abc	60742 ± 19025 abc	42243 ± 13735 abc	40707 ± 18961 abc	82615 ± 24632 a	72259 ± 22774 abc
1-pentanol	1258	55	59359 ± 12221 abc	65562 ± 7545 ab	59980 ± 12298 abc	78033 ± 14052 a	30267 ± 9581 c	69650 ± 15728 a	69468 ± 12153 a
2,3-dimethyl 1 butanol	1290	84	2342 ± 330 a	3659 ± 2560 a	4472 ± 2379 a	3392 ± 2215 a	5074 ± 3242 a	4793 ± 2124 a	4803 ± 2695 a
2-butanone	905	43	24949 ± 7359 b	40779 ± 11964 b	29027 ± 10898 b	3138 ± 1423 c	29856 ± 10334 b	24647 ± 8979 bc	70884 ± 5837 a
2-ethyl hexanol	1493	57	88605 ± 8066 a	81751 ± 14208 a	85988 ± 12042 a	77512 ± 10987 a	81287 ± 12234 a	77151 ± 8256 a	73060 ± 9452 a
2-heptanone	1171	43	489387 ± 31968 c	985743 ± 139997 b	527973 ± 24413 c	89184 ± 25485 d	2164449 ± 168494 a	422801 ± 64116 c	557404 ± 13566 c
2-heptenal	1311	55	3802 ± 946 ab	11084 ± 4944 a	8404 ± 3461 ab	3760 ± 2198 ab	10769 ± 6346 a	5795 ± 1851 ab	9900 ± 2629 a
2-methylbutanal	915	41	58223 ± 8922 bcd	63807 ± 13894 bc	69153 ± 16120 ab	49383 ± 11175 bcd	96446 ± 16025 a	38135 ± 3055 cd	29886 ± 6408 d
2-nonanone	1378	43	307905 ± 17807 cd	1293427 ± 203419 a	307092 ± 26899 cd	74311 ± 4297 d	943441 ± 121250 b	104870 ± 49145 d	386275 ± 43938 c
2-nonen-4-one	1471	69	8580 ± 2408 bc	15787 ± 7202 ab	6006 ± 2819 bc	2020 ± 879 c	24529 ± 11086 a	2756 ± 2358 bc	4813 ± 2402 bc
2-octanone	1274	58	96039 ± 38416 a	42712 ± 29558 ab	37512 ± 36879 ab	2238 ± 578 b	25386 ± 17332 b	9795 ± 8002 b	484 ± 484 b
2-Pentanone, 4-hydroxy-4-methyl-	1361	43	38570 ± 12200 a	8447 ± 4885 abc	5995 ± 1812 bc	37155 ± 23244 ab	12923 ± 3896 abc	10704 ± 4670 abc	5235 ± 2377 c
2-undecanone	1591	58	201865 ± 8351 c	361157 ± 12458 a	202983 ± 16662 c	34652 ± 2313 e	281119 ± 55535 b	93398 ± 4957 d	233836 ± 5813 bc

[a] retention index calculated, and [b] selected ion (*m*/*z*) used for integration. Number followed by different letters in the same row are significantly different ($P<0.05$).

Supplementary Table S1 cont. Abundance of aroma metabolites in fermented milk samples. (Mean abundance ± standard deviation).

Compound	RI[a]	Ions[b]	Fermented milk samples						
			BL1	CH-1	CL3	CL3ST	LH	ST	YF-L811
3_methyl_3_buten_1_ol	1253	56	5484 ± 340 cd	16908 ± 1390 a	7531 ± 1293 bc	2674 ± 436 cd	13058 ± 5901 ab	15302 ± 449 a	12441 ± 1823 ab
3-methylbutanol	1217	55	17129 ± 2262 b	15095 ± 8379 b	6866 ± 5352 bc	46692 ± 4502 a	46204 ± 3154 a	8172 ± 6529 bc	1719 ± 135 c
3-penten-2-one	1129	69	17579 ± 1675 bc	32337 ± 7127 b	20305 ± 4594 b	4576 ± 1382 c	78592 ± 10760 a	18022 ± 6039 bc	22856 ± 6627 b
4-methyl 2-heptanone	1196	43	9399 ± 1798 b	73054 ± 32164 a	40893 ± 22770 ab	7684 ± 1172 b	43065 ± 38196 ab	10361 ± 2048 b	31142 ± 17570 ab
4-nonanone	1313	43	9934 ± 4068 ab	12818 ± 1815 ab	10504 ± 3876 ab	4459 ± 3504 b	18395 ± 4710 a	12419 ± 1744 ab	10895 ± 4074 ab
acetaldehyde	587	44	271543 ± 8757 d	1147796 ± 36292 a	310891 ± 24782 d	102577 ± 39398 e	1133213 ± 43475 a	429395 ± 33365 c	777916 ± 50934 b
acetic acid	1451	60	2923877 ± 33729 a	1288857 ± 253093 cd	2845126 ± 176684 a	1982171 ± 773361 bc	2599603 ± 220783 ab	450601 ± 10305 e	774650 ± 71042 d
acetoin	1280	45	2886230 ± 1086177 b	3148620 ± 52123 b	2613484 ± 1343048 b	54636 ± 8569 c	122203 ± 23994 c	5559282 ± 136073 a	3417312 ± 74267 b
acetone	724	43	100708 ± 5916 b	88780 ± 18908 b	117815 ± 26206 b	20136 ± 4104 c	162259 ± 22922 a	92173 ± 18170 b	99259 ± 14919 b
benzaldehyde	1507	106	694045 ± 116511 a	308813 ± 51182 bc	495281 ± 70632 ab	446661 ± 117333 b	287314 ± 27473 bc	690612 ± 125956 a	180192 ± 45531 c
benzoic acid	2433	105	694113 ± 9130 ab	804070 ± 146304 a	675629 ± 35100 ab	107599 ± 13272 d	595837 ± 30191 bc	128398 ± 3353 d	484629 ± 8811 c
butanoic acid	1627	60	665885 ± 14054 a	683731 ± 52638 a	672860 ± 21361 a	160626 ± 44562 c	533799 ± 22688 b	687644 ± 38965 a	671660 ± 15485 a
decanoic acid	2246	60	488391 ± 28605 b	481010 ± 6176 b	489374 ± 20235 b	370333 ± 53027 c	707562 ± 8617 a	699896 ± 12575 a	523699 ± 21564 b
diacetyl	970	86	371380 ± 55121 d	548282 ± 19771 c	319415 ± 53212 d	20018 ± 9973 e	131090 ± 37811 e	1560465 ± 117423 a	803500 ± 81437 b
diisoamyl	912	99	21546 ± 4194 ab	31416 ± 6200 a	32918 ± 10793 a	31490 ± 17021 a	34860 ± 17897 a	33672 ± 11291 a	30704 ± 14532 ab

[a] retention index calculated, and [b] selected ion (*m*/*z*) used for integration. Number followed by different letters in the same row are significantly different ($P<0.05$).

Supplementary Table S1 cont. Abundance of aroma metabolites in fermented milk samples. (Mean abundance ± standard deviation).

Compound	RI[a]	Ions[b]	Fermented milk samples						
			BL1	CH-1	CL3	CL3ST	LH	ST	YF-L811
dimethyl disulfide	1059	94	65429 ± 7433 c	82127 ± 26569 c	57461 ± 6977 c	36848 ± 8578 e	277725 ± 16324 a	62410 ± 9267 c	53928 ± 21509 c
dimethyl trisulfide	1346	126	28923 ± 2764 bc	38891 ± 3441 b	29077 ± 3305 bc	13439 ± 1791 bc	102431 ± 3085 a	36686 ± 2622 b	28998 ± 1588 bc
ethanol	938	45	6385164 ± 972519 a	6002559 ± 1707281 a	5305498 ± 1954357 ab	7750165 ± 3261255 a	3788337 ± 789409 abc	3802049 ± 1259647 abc	5847477 ± 1698689 a
ethyl acetate	892	43	53643 ± 2872 b	109489 ± 39031 b	39892 ± 4413 b	2290747 ± 763567 a	318401 ± 135038 b	32667 ± 8763 b	33846 ± 16092 b
ethyl octanoate	1425	88	99137 ± 24290 b	216603 ± 89901 b	94101 ± 34917 b	720981 ± 302647 a	43923 ± 15086 b	151255 ± 63019 b	153003 ± 50482 b
ethyl decanoate	1634	88	146816 ± 31723 b	106116 ± 41831 b	136298 ± 49883 b	821074 ± 391659 a	46023 ± 12042 b	139609 ± 62747 b	81237 ± 25985 b
heptanal	1169	70	31464 ± 6200 bc	53345 ± 23946 abc	22618 ± 11310 c	769 ± 522 c	24802 ± 11428 c	105240 ± 24518 a	30656 ± 15328 bc
heptanoic acid	1946	60	54193 ± 2351 ab	58011 ± 3445 a	54494 ± 3012 ab	14704 ± 2802 c	51689 ± 1256 ab	54639 ± 3391 ab	56656 ± 820 a
hexanal	1079	44	50363 ± 9981 cd	79602 ± 36729 bc	47052 ± 7198 cd	3141 ± 887 d	93687 ± 8599 bc	174872 ± 33457 a	112241 ± 39813 abc
hexanoic acid	1846	60	1916996 ± 27681 abc	2116981 ± 143881 a	1901586 ± 27292 bc	434019 ± 114530 d	1801487 ± 45924 c	2030737 ± 99701 ab	2109575 ± 38033 a
hexanol	1363	56	63951 ± 12548 a	72853 ± 18725 a	66516 ± 13768 a	85653 ± 18583 a	59641 ± 7451 a	83696 ± 24713 a	70881 ± 16125 a
Isobutyric acid	1566	43	34198 ± 6370 a	31207 ± 5134 a	31788 ± 5815 a	27016 ± 4815 a	34093 ± 5525 a	25123 ± 6217 a	27215 ± 6884 a
isovaleric acid	1669	60	31721 ± 3133 b	57350 ± 6772 a	29518 ± 3218 bc	61291 ± 24848 a	60124 ± 2402 a	6509 ± 956 c	15850 ± 1366 bc
methional	1444	48	0 ± 0 c	2583 ± 1468 b	0 ± 0 c	0 ± 0 c	5110 ± 888 a	0 ± 0 c	796 ± 796 c
methylpyrazine	1264	94	22538 ± 2691 b	0 ± 0 d	19105 ± 1303 c	19262 ± 1087 c	29787 ± 1053 a	0 ± 0 d	0 ± 0 d

[a] retention index calculated, and [b] selected ion (*m*/*z*) used for integration. Number followed by different letters in the same row are significantly different (P<0.05).

Supplementary Table S1 cont. Abundance of aroma metabolites in fermented milk samples. (Mean abundance ± standard deviation).

Compound	RI[a]	Ions[b]	Fermented milk samples						
			BL1	CH-1	CL3	CL3ST	LH	ST	YF-L811
nonanoic acid	2146	60	102749 ± 33370 a	64243 ± 9819 ab	46757 ± 4423 bc	23448 ± 3122 c	82554 ± 4217 ab	44541 ± 1417 bc	66880 ± 15699 ab
octanoic acid	2046	60	1872047 ± 65063 b	2100197 ± 95864 a	1833061 ± 83059 b	544455 ± 142845 c	1900534 ± 18693 b	1843421 ± 53191 b	1965655 ± 12129 ab
propionic acid	1538	74	52393 ± 796 a	10543 ± 592 b	49664 ± 1881 a	9385 ± 2864 b	11187 ± 545 b	8296 ± 508 b	9789 ± 390 b

[a] retention index calculated, and [b] selected ion (*m*/*z*) used for integration. Number followed by different letters in the same row are significantly different ($P<0.05$).
